# Associations Between Lumbar Vertebral Bone Marrow and Paraspinal Muscle Fat Compositions—An Investigation by Chemical Shift Encoding-Based Water-Fat MRI

**DOI:** 10.3389/fendo.2018.00563

**Published:** 2018-09-28

**Authors:** Nico Sollmann, Michael Dieckmeyer, Sarah Schlaeger, Alexander Rohrmeier, Jan Syvaeri, Maximilian N. Diefenbach, Dominik Weidlich, Stefan Ruschke, Elisabeth Klupp, Daniela Franz, Ernst J. Rummeny, Claus Zimmer, Jan S. Kirschke, Dimitrios C. Karampinos, Thomas Baum

**Affiliations:** ^1^Department of Diagnostic and Interventional Neuroradiology, Klinikum rechts der Isar, Technische Universität München, Munich, Germany; ^2^TUM-Neuroimaging Center, Klinikum rechts der Isar, Technische Universität München, Munich, Germany; ^3^Department of Diagnostic and Interventional Radiology, Klinikum rechts der Isar, Technische Universität München, Munich, Germany

**Keywords:** bone marrow, chemical shift encoding-based water-fat imaging, lumbar spine, muscle fat, paraspinal musculature, proton density fat fraction

## Abstract

**Purpose:** Advanced magnetic resonance imaging (MRI) methods enable non-invasive quantification of body fat situated in different compartments. At the level of the lumbar spine, the paraspinal musculature is the compartment spatially and functionally closely related to the vertebral column, and both vertebral bone marrow fat (BMF) and paraspinal musculature fat contents have independently shown to be altered in various metabolic and degenerative diseases. However, despite their close relationships, potential correlations between fat compositions of these compartments remain largely unclear.

**Materials and Methods:** Thirty-nine female subjects (38.5% premenopausal women, 29.9 ± 7.1 years; 61.5% postmenopausal women, 63.2 ± 6.3 years) underwent MRI at 3T of the lumbar spine using axially- and sagittally-prescribed gradient echo sequences for chemical shift encoding-based water-fat separation. The erector spinae muscles and vertebral bodies of L1–L5 were segmented to determine the proton density fat fraction (PDFF) of the paraspinal and vertebral bone marrow compartments. Correlations were calculated between the PDFF of the paraspinal muscle and bone marrow compartments.

**Results:** The average PDFF of the paraspinal muscle and bone marrow compartments were significantly lower in premenopausal women when compared to postmenopausal women (11.6 ± 2.9% vs. 24.6 ± 7.1% & 28.8 ± 8.3% vs. 47.2 ± 8.5%; *p* < 0.001 for both comparisons). In premenopausal women, no significant correlation was found between the PDFF of the erector spinae muscles and the PDFF of the bone marrow of lumbar vertebral bodies (*p* = 0.907). In contrast, a significant correlation was shown in postmenopausal women (*r* = 0.457, *p* = 0.025). Significance was preserved after inclusion of age and body mass index (BMI) as control variables (*r* = 0.472, *p* = 0.027).

**Conclusion:** This study revealed significant correlations between the PDFF of paraspinal and vertebral bone marrow compartments in postmenopausal women. The PDFF of the paraspinal and vertebral bone marrow compartments and their correlations might potentially serve as biomarkers; however, future studies including more subjects are required to evaluate distinct clinical value and reliability. Future studies should also follow up our findings in patients suffering from metabolic and degenerative diseases to clarify how these correlations change in the course of such diseases.

## Introduction

Vertebral bone marrow, located in the cavities of trabecular bone and consisting predominantly of adipocytes (yellow marrow regions) or adipocytes and hematopoietic red blood cells (red marrow regions), plays a considerable role for bone health. Various metabolic diseases have shown to be associated with changes in fat compositions of vertebral bone marrow in humans. For instance, patients with osteoporosis showed significantly increased bone marrow fat (BMF) fractions when compared to healthy subjects, which suggests a shift of differentiation of mesenchymal stem cells to adipocytes rather than to osteoblasts (1, 2). Furthermore, patients suffering from type 2 diabetes mellitus (T2DM) showed significantly lower BMF unsaturation levels, and high HbA1c levels were associated with elevated BMF (3–5). In anorexia nervosa, vertebral BMF contents demonstrated to be increased (6). Importantly, the finding of elevated BMF in metabolic diseases has clinical relevance: alterations of bone marrow adiposity have already been associated with bone weakness and prevalent vertebral fractures (7–10).

Furthermore, metabolic diseases have also demonstrated independently to have an impact on other compartments of the body containing fat including paraspinal musculature, which is the compartment spatially and functionally closely related to the vertebral column, thus being particularly important for spine stabilization, balance keeping, and mobility (11, 12). In this context, T2DM, degenerative changes, low back pain (LBP), neuromuscular diseases (NMDs), and injuries, amongst others, have demonstrated to entail transformation of muscle tissue (13–18). Furthermore, osteoporosis, mainly known as a bone disease characterized by low bone mass, has also shown to have associations with changes in the composition of paraspinal musculature in terms of increased muscle fat infiltration (MFI), thus suggesting close interactions between vertebrae and paraspinal muscles (19). However, evidence of such interaction is very scarce. Increased paraspinal MFI principally harbors negative implications on muscle function and movement (11, 20, 21).

Regarding the assessment of the body's fat compositions, various imaging techniques including dual energy X-ray absorptiometry (DXA), computed tomography (CT), and magnetic resonance imaging (MRI) are available to date. DXA can differentiate bone, fat, and lean soft tissue, while CT also enables determinations of the amount of fat depositions and adipose tissue volumes (22–24). MRI enables both qualitative and quantitative evaluation without ionizing radiation when compared to DXA and CT, and it makes the evaluation of more sophisticated parameters possible, which include assessments of regional body fat distributions and fat phenotyping (25, 26). Up to now, most studies investigated patho-anatomical features by means of conventional T1-weighted or T2-weighted MRI, thus primarily using qualitative analyses. However, advanced MRI-based techniques allowing quantitative assessments have been introduced, such as magnetic resonance spectroscopy (MRS) and chemical shift encoding-based water-fat MRI (25–27). Chemical shift encoding-based water-fat MRI represents a time-efficient, field map-insensitive technique to determine fat and water signal of the human body, thus making non-invasive quantification of water-fat compositions possible (28–32). In this context, determination of the proton density fat fraction (PDFF) derived from chemical shift encoding-based water-fat MRI is a reliable approach to extract quantitative information about fat compositions of different body compartments, with good concordance between PDFF measurements and histology or MRS (33, 34).

Despite its evident close spatial and functional relationships, evidence for interactions between lumbar vertebral body and paraspinal muscle fat compositions, investigated *in-vivo* by means of non-invasive, quantitative imaging, is mostly lacking. However, it is now technically possible to determine the PDFF of different compartments by means of chemical shift encoding-based water-fat MRI derived from the same imaging sequences, allowing intra-subject correlations. Thus, the present study systematically investigates the correlation between vertebral body and paraspinal muscle PDFF in a cohort of female subjects without spinal pathology.

## Materials and methods

### Study population

This study was approved by the Institutional Review Board. All subjects provided written informed consent prior to study inclusion.

Overall, 39 subjects were recruited. Inclusion criteria were age above 18 years, female gender, clear pre- or postmenopausal status based on a self-reported questionnaire, and physiological spine anatomy according to clinical examination and MRI. Exclusion criteria were any history of vertebral body fractures, any history of high-performance sports, LBP, NMD, anorexia nervosa, T2DM, or other metabolic diseases (except for osteopenia or osteoporosis), and general MRI contraindications. Enrolled subjects were divided into premenopausal and postmenopausal women for data analyses. The body height and weight of each subject was documented for the calculation of the body mass index (BMI), which was obtained by dividing the individual body weight by height squared.

Fifteen subjects (38.5%) were categorized as young, premenopausal women, whereas the remaining 24 subjects (61.5%) consisted of postmenopausal women. Within 1 month in relation to study inclusion, 22 postmenopausal women (91.7%) underwent a clinically indicated DXA exam (Prodigy, GE Healthcare, Chalfont St Giles, Buckinghamshire, UK), which was done in addition to the MRI scanning performed for this study's purposes. According to the resulting T-scores, 72.7% of these postmenopausal women had a normal bone status, while 18.2% and 9.1% of these women were classified as osteopenic and osteoporotic, respectively.

### Magnetic resonance imaging

MRI was performed at 3T on the same scanner in all enrolled subjects (Ingenia, Philips Healthcare, Best, Netherlands). Subjects were positioned head-first in a supine position. During image acquisition, we used a built-in 12-channel posterior coil, a 16-channel anterior coil, and a whole-body coil.

For chemical shift encoding-based water-fat separation at the level of the lumbar spine, two sequences were acquired. For the later assessment of the paraspinal musculature, an axially-prescribed six-echo sequence was acquired with the following parameters: repetition time (TR)/echo time (TE)/ΔTE = 6.4/1.1/0.8 ms, voxel size = 3.2 × 2.0 × 4.0 mm3, field of view (FOV) = 220 × 401 × 252 mm^3^, frequency encoding direction = L/R, scan time = 1 min and 25 s. Regarding the assessment of bone marrow of vertebral bodies, a sagittally-prescribed eight-echo sequence was acquired with the following sequence adjustments: TR/TE/ΔTE = 11/1.4/1.1 ms, voxel size = 1.8 × 1.8 × 4.0 mm3, FOV = 220 × 220 × 80 mm3, frequency encoding direction = A/P (to minimize breathing artifacts), scan time = 1 min and 17 s. Both sequences acquired the echoes in a single TR using bipolar readout gradients. To reduce T1 bias effects, a flip angle of 3° was applied (28, 35).

### Fat quantification and image segmentation

First, the MRI data were processed online using the vendor's routines. Phase error correction and a complex-based water-fat decomposition considering a precalibrated seven-peak fat spectrum and a single T2^*^ were performed within the context of the multi-echo mDIXON algorithm. The imaging-based PDFF maps were then the result of defining the ratio of the fat signal over the sum of fat and water signals.

Within the PDFF maps of each subject, manual regions of interest (ROIs) were carefully placed using MITK ([http://mitk.org/wiki/The_Medical_Imaging_Interaction_Toolkit_(MITK)]; German Cancer Research Center, Division of Medical and Biological Informatics, Medical Imaging Interaction Toolkit, Heidelberg, Germany). This was achieved separately for the following seven structures: right erector spinae muscle (ESR), left erector spinae muscle (ESL), vertebral bodies L1, L2, L3, L4 and L5. Segmentations were performed by a radiologist.

**Figure 1:**
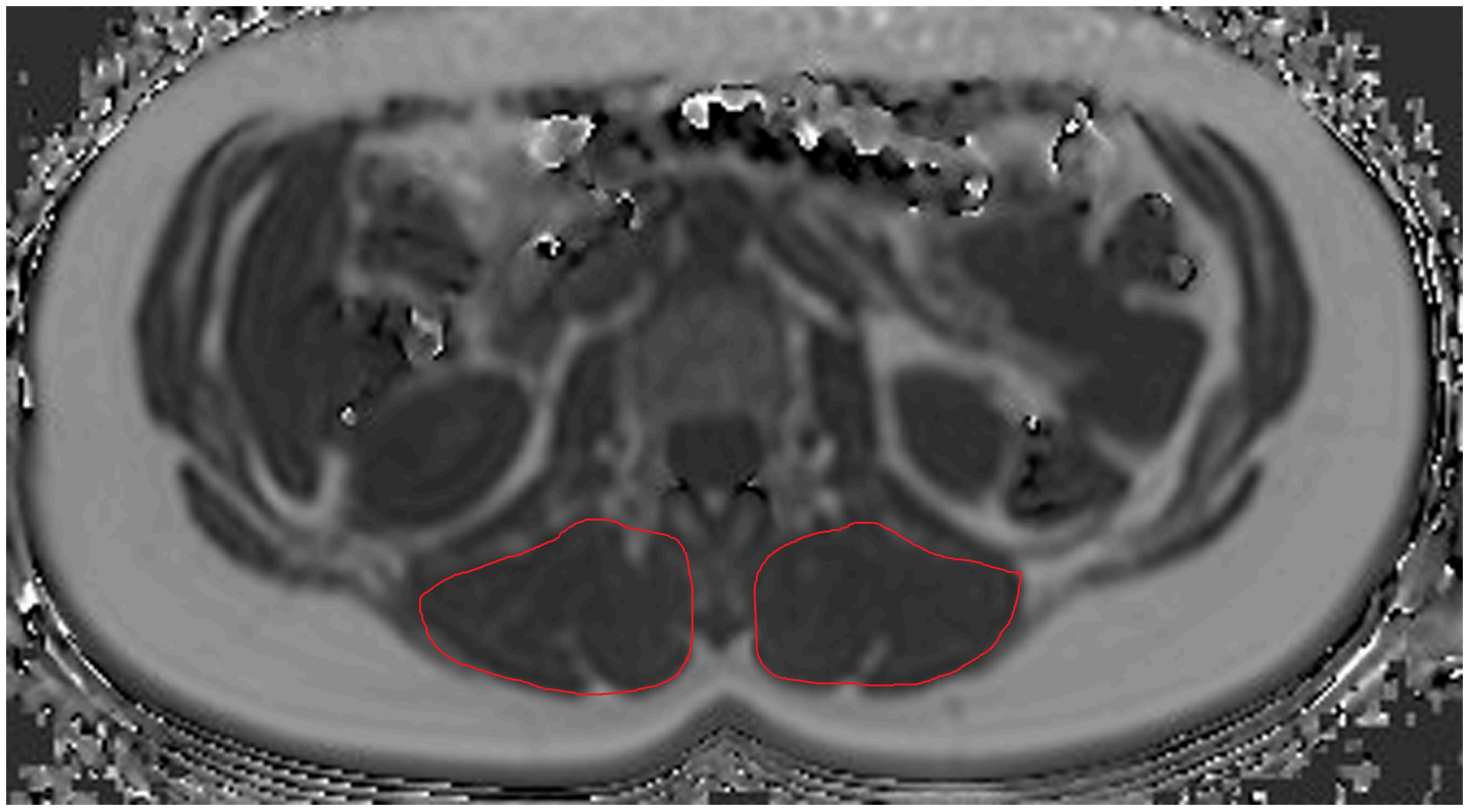
Region of interest (ROI) placement in the paraspinal compartment. Representative segmentation of the left erector spinae muscle (ESL) and right erector spinae muscle (ESR) in the proton density fat fraction (PDFF) map of a 39-year-old woman.

**Figure 2:**
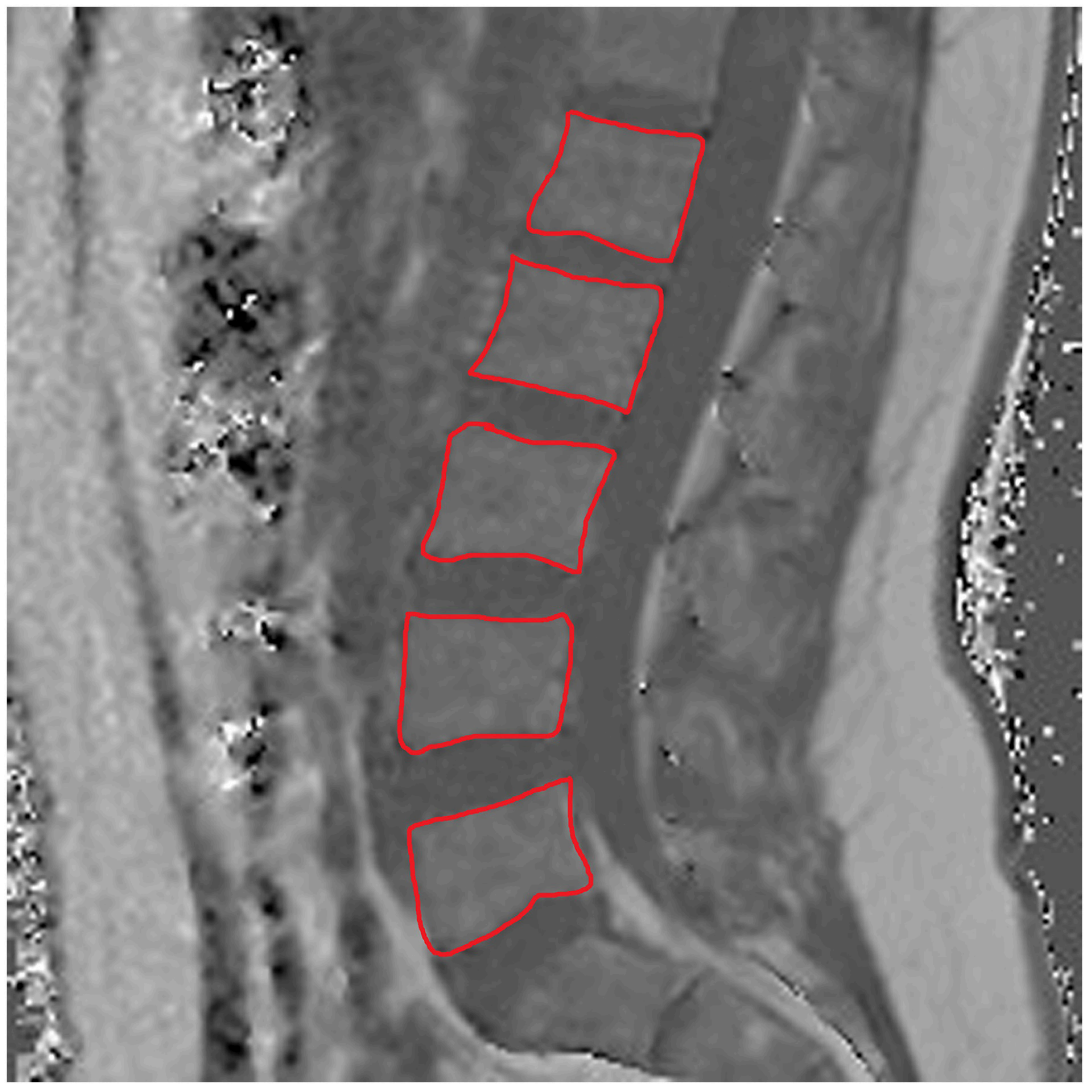
Region of interest (ROI) placement in the bone marrow compartment. Representative segmentation of lumbar vertebral bodies 1–5 (L1–L5) in the proton density fat fraction (PDFF) map of a 25-year-old woman.

Concerning ROI drawing in the ESR and ESL, images derived from the axially-prescribed six-echo sequence were separately segmented from the upper endplate level of L2 to the lower endplate level of L5, with ROIs being placed at the muscle contour whilst avoiding the inclusion of subcutaneous fat or the muscle-fat interface (Figure 1). Regarding ROI definition in the vertebral bodies, images stemming from the sagittally-prescribed sequence were opened to place the ROIs in each sagittal slice for L1–L5 (Figure 2). The posterior elements and any sclerotic changes of the endplates were excluded.

Subsequent to ROI placements, PDFFs were extracted by averaging the values derived from the single ROIs placed in the consecutive slices. Furthermore, the PDFFs of the ESR and ESL were averaged to obtain one PDFF value per subject regarding the paraspinal muscle compartment. Analogously, the vertebral PDFFs of L1/L2, L2/L3, L3/L4, L4/L5, and L1–L5 were also averaged, resulting in segment-specific and total lumbar PDFF measurements for the bone marrow compartments per subject.

### Statistical analyses

SPSS (version 20.0; IBM SPSS Statistics for Windows, Armonk, NY, USA) and GraphPad Prism (version 6.04; GraphPad Software Inc., La Jolla, CA, USA) were used for statistical data analyses and graph generation. For all statistical tests, the level of significance was set at *p* < 0.05 (two-sided).

First, descriptive statistics including mean ± standard deviation (SD), median, minimum, and maximum were calculated for subject-related characteristics and the PDFF measurements of the paraspinal and bone marrow compartments, which was performed separately for pre- and postmenopausal women. To compare premenopausal vs. postmenopausal women in terms of subject characteristics and obtained PDFF measurements, Mann-Whitney tests were applied. Furthermore, coefficients of variation (CVs) were calculated as measures for dispersion regarding the PDFF measurements. Then, Pearson correlations were calculated considering age, BMI, PDFFs of the paraspinal muscle compartment, and PDFFs of the bone marrow compartment (segment-specific and total vertebral measurements), which was again achieved for pre- and postmenopausal women, respectively. Partial correlations with age and BMI as control variables were added to further investigate the correlations between the PDFF of the paraspinal and bone marrow compartments.

## Results

### Enrolled subjects

MRI with chemical shift encoding-based water-fat separation was successfully acquired in all enrolled subjects. There was a statistically significant difference between both groups (pre- and postmenopausal women) in terms of age (premenopausal women: 29.9 ± 7.1 years, range: 21–42 years; postmenopausal women: 63.2 ± 6.3 years, range: 54–78 years; *p* < 0.001), but not regarding BMI (premenopausal women: 26.0 ± 1.6 kg/m^2^, range: 23.7–28.4 kg/m^2^; postmenopausal women: 25.7 ± 4.2 kg/m^2^, range: 18.8–35.3 kg/m^2^; *p* = 0.573).

### Measurements of the PDFF

**Figure 3:**
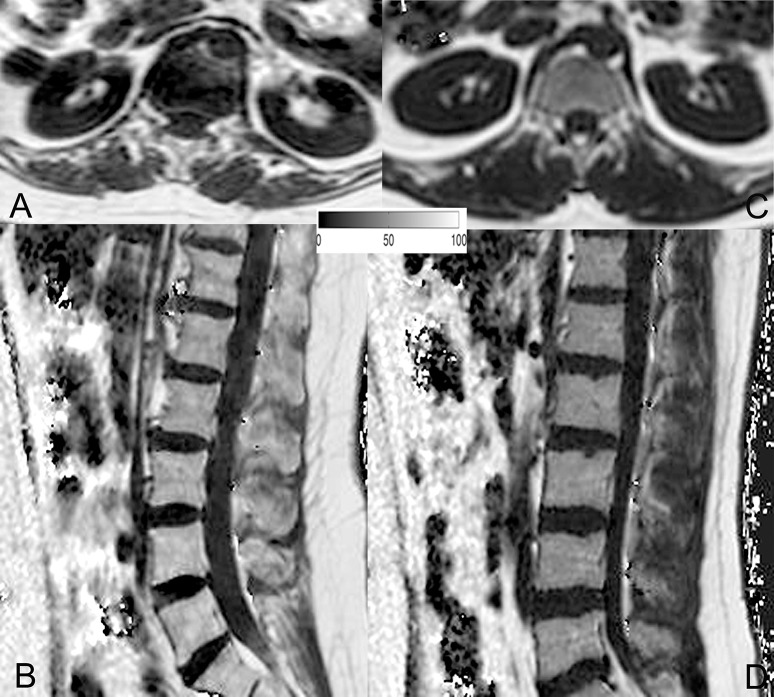
Representative proton density fat fraction (PDFF) maps. Representative axial and sagittal PDFF maps of the erector spinae muscles and the lumbar bone marrow of a postmenopausal woman [**(A,B)**; age: 59 years; mean erector spinae muscle PDFF: 6.1%; mean L1–L5 bone marrow PDFF: 53.6%] and a premenopausal woman [**(C,D)**; age: 28 years; mean erector spinae muscle PDFF: 4.5%; mean L1–L5 bone marrow PDFF: 42.7%] are shown in this figure.

The average PDFF of the erector spinae muscles was 11.6 ± 2.9% (range: 5.2–15.6%) in premenopausal women and 24.6 ± 7.1% (range: 16.5–43.7%) in postmenopausal women (*p* < 0.001). Thus, we observed a CV of 24.3% in premenopausal women and a CV of 28.0% in postmenopausal women. Furthermore, in premenopausal women, the PDFF of the bone marrow of lumbar vertebral bodies was 28.8 ± 8.3% (range: 18.7–45.9%), whereas it was 47.2 ± 8.5% (range: 30.7–61.8%) in postmenopausal subjects (*p* < 0.001). Correspondingly, PDFF measurements showed a CV of 27.7% in premenopausal women and a CV of 17.6% in postmenopausal women. Representative PDFF maps of the erector spinae muscles and lumbar bone marrow derived from pre- and postmenopausal women are shown in Figure 3.

### Correlations between the PDFF of the paraspinal and bone marrow compartments

In the cohort of premenopausal women, no statistically significant correlation was found for the PDFF of the erector spinae muscles and the PDFF of the bone marrow of lumbar vertebral bodies (*p* = 0.907). Correspondingly, partial correlation considering age and BMI as control variables did not result in a significant correlation either (*p* = 0.760).

In contrast to premenopausal women, a significant correlation between the PDFF of the paraspinal and bone marrow compartments was observed in postmenopausal women (L1–L5: *r* = 0.457, *p* = 0.025; Table 1). Furthermore, significant correlations were also revealed between the PDFF of the paraspinal compartment and different spinal segments (L1/L2: *r* = 0.422, *p* = 0.040; L2/L3: *r* = 0.502, *p* = 0.012; L3/L4: *r* = 0.496, *p* = 0.014; L4/L5: *r* = 0.406, *p* = 0.049). After inclusion of age and BMI as control variables, statistical significance was preserved for partial correlation (L1–L5: *r* = 0.472, *p* = 0.027).

**Table 1:** Correlations in postmenopausal women.

		**Age (in years)**	**BMI (in kg/m^2^)**	**PDFF (in %) paraspinal compartment**	**PDFF (in %) bone marrow compartment**
**Age (in years)**	Pearson correlation coefficient	1	−0.126	0.572	0.120
	*p*	–	n.s.	0.004	n.s.
**BMI (in kg/m**^2^**)**	Pearson correlation coefficient	−0.126	1	0.053	0.063
	*p*	n.s.	–	n.s.	n.s.
**PDFF (in %) paraspinal compartment**	Pearson correlation coefficient	0.572	0.053	1	0.457
	*p*	0.004	n.s.	–	0.025
**PDFF (in %) bone marrow compartment**	Pearson correlation coefficient	0.120	0.063	0.457	1
	*p*	n.s.	n.s.	0.025	–

*This table illustrates the results of the correlation of age, body mass index (BMI), proton density fat fraction (PDFF) measurements of the paraspinal compartment, and PDFF measurements of the bone marrow compartment. Regarding the PDFF of the paraspinal compartment, the left erector spinae muscle (ESL) and right erector spinae muscle (ESR) were considered from the upper endplate level of L2 to the lower endplate level of L5. The PDFF of the bone marrow compartment was derived from measurements in vertebral bodies L1–L5. Pearson correlation coefficients are provided for each correlation, and p-values indicate statistical significance of the respective correlation (n.s.: not significant)*.

**Figure 4:**
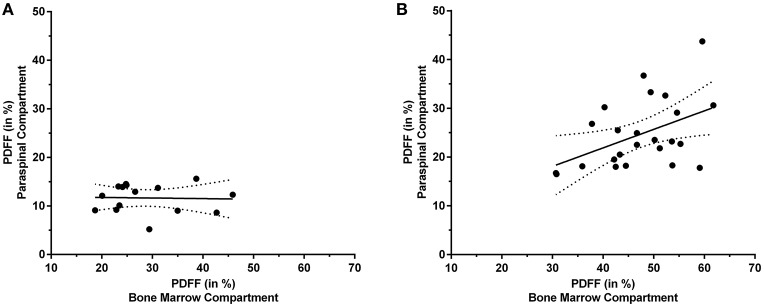
Proton density fat fraction (PDFF) of the paraspinal and bone marrow compartments. This figure plots the PDFF measurements of the paraspinal against the PDFF measurements of the bone marrow compartments for premenopausal women [**(A)**; *y* = −0.012^*^x + 11.970] and postmenopausal women [**(B)**; *y* = 0.380^*^x + 6.695], respectively. The areas between the dotted lines represent the 95% confidence band of the best-fit line.

Figure 4 plots the obtained PDFFs of the paraspinal against the bone marrow compartments for pre- and postmenopausal women, respectively.

## Discussion

This study used chemical shift encoding-based water-fat MRI at the lumbar spine to extract PDFF measurements of paraspinal muscles (ESR and ESL) and vertebral bodies (L1–L5) in pre- and postmenopausal women. An age-dependent correlation between the PDFF of the paraspinal and bone marrow compartments was observed, with postmenopausal women showing statistically significant correlations even after consideration of age and BMI as control variables. Although several diseases have independently demonstrated to affect vertebral body or paraspinal muscle fat compositions, interactions between these compartments have not yet been investigated by means of advanced quantitative imaging in subjects without spinal diseases. However, close spatial and functional relationships between paraspinal musculature and the vertebral column are evident, most prominently reflected by major interactions in terms of body movement, spine stabilization, and balance keeping (11, 12).

Subjects diagnosed with osteoporosis have shown to harbor significantly elevated BMF when compared to healthy individuals (25, 26, 36, 37). Griffith et al. used MRS to investigate associations between vertebral bone marrow fat and osteoporosis, with significantly increased BMF in osteoporotic subjects (1, 2). More recently, Kuhn et al. were able to show that the PDFF was greater in osteoporotic than in healthy vertebrae (38). It has also been shown repeatedly that with increasing age, especially females are prone to accelerated fatty conversion of the bone marrow compared to males, with the menopause potentially representing the main trigger (39–42). The finding of reduced bone mineral density and high incidences of vertebral fragility fractures particularly among postmenopausal women on the one hand and clear changes in BMF on the other hand suggests strong correlations between vertebral bone marrow composition and osteoporosis (37, 43, 44). In addition, osteoporosis has also demonstrated to have associations with changes in composition of paraspinal musculature: Kim et al. used MRI in postmenopausal women, showing that vertebral fractures were associated with profound changes of paraspinal musculature, represented by reductions of the cross sectional area and increased MFI (19). The study of Kim et al. applied semi-quantitative measurements with three visual scale grades (19).

Taken together, especially postmenopausal women seem to be predisposed for investigations of interactions between vertebrae and paraspinal musculature. Indeed, we found significant correlations between the PDFF of paraspinal muscles and vertebral bodies in our cohort. Furthermore, in contrast to the investigation of Kim et al., correlations were observed using more advanced, high-resolution MRI with chemical shift encoding-based water-fat separation, thus allowing multi-segmental evaluations of the PDFF, a parameter that has not been evaluated in their study (19). More importantly, results were obtained in postmenopausal women without vertebral fractures, thus providing first evidence for associations between vertebral body and paraspinal muscle fat compositions in subjects without any history of spinal fractures.

Other conditions have also proven impact on bone marrow composition (25, 26, 36). In T2DM, vertebral BMF was comparable between diabetic postmenopausal women and healthy subjects; however, mean unsaturation levels were significantly lower in subjects diagnosed with T2DM, and significant correlations of vertebral BMF content with adjusted visceral adipose tissue and HbA1c were observed in these subjects (3). Similarly, T2DM in postmenopausal women was associated with lower unsaturation and higher saturation levels, with diabetic subjects having fragility fractures showing the lowest marrow unsaturation and highest saturation (4). Furthermore, degenerative changes at the spine and LBP demonstrated relationships to alterations in muscle composition, with paraspinal MFI being associated with LBP, and disc degeneration and paraspinal muscle atrophy being positively correlated (14, 16). Again, correlations of the paraspinal muscle and bone marrow compartments in terms of quantitative BMF evaluations have not been routinely achieved yet. Significant positive correlations between vertebral body and paraspinal muscle PDFF measurements, as observed in the present study among postmenopausal women, might also be observed in these conditions, with the PDFF then potentially serving as an easily assessable, non-invasive biomarker of clinically relevant changes in fat content. However, future studies enrolling patients with such diseases have to clarify the role of correlations between PDFF measurements derived from different fat compartments at the level of the lumbar spine.

As aforementioned, we found a significant correlation between the PDFF of the paraspinal and bone marrow compartments in postmenopausal women, but not in young, premenopausal women. The reason for this finding might be related to the observation that for premenopausal women, the PDFF ranges were relatively small, thus indicating rather homogeneous PDFF distributions in young women. In contrast, the PDFF ranges were considerably wider in postmenopausal women, most probably reflecting more distinct changes of fat compositions of both paraspinal musculature and vertebral bone marrow. In this context, it has already been revealed that a relevant age-dependent increase in fatty conversion is present particularly in women (39–42). However, exploration of the distinct biological causes for the observed results goes beyond the scope of this imaging study and has to be the purpose of future investigations.

When interpreting the results of the present study, we also have to bear some limitations in mind. First, the comparatively small cohort size has to be regarded as a main limitation. Thus, the results presented have to be interpreted with caution and should be confirmed by follow-up studies enrolling more subjects. In this context, the absence of a statistically significant correlation in young, premenopausal women could be related to the comparatively low number of subjects assigned to this group. Hence, future studies might focus on larger cohorts including particularly more premenopausal women to clarify whether the subtler changes of vertebral body and paraspinal muscle fat compositions in younger years truly do not show significant correlations. Second, the enrolment of women only might be regarded as a limitation, and further studies might explore potential correlations of PDFF measurements among men. Previous studies indicated clear sex differences in terms of vertebral body and paraspinal muscle fat compositions (39–42). Therefore, separate, sex-specific analyses seem justified, with this study focusing on women first. The results of the present study provide a good basis for further exploration of PDFF correlations in both sexes. Third, we did not include subjects suffering from metabolic diseases such as T2DM. As a consequence, upcoming investigations may distinctly explore correlations in pathological conditions to determine whether vertebral body and paraspinal muscle PDFFs and their correlations might serve as biomarkers in the clinical setting. Additionally, interventions should be performed to evaluate whether physical exercise or medication, for instance, might have an effect upon the correlations between vertebral body and paraspinal muscle PDFF measurements. Effects have been observed regarding the one or the other compartment in a limited amount of studies so far (45–47). Moreover, it would be interesting to further explore the distribution of lipids within the paraspinal muscles and to distinctly quantify the intramyocellular lipid and extramyocellular lipid levels. However, such investigations would require the acquisition of MRS, which was not available for the present study.

## Conclusion

Using chemical shift encoding-based water-fat MRI at the lumbar spine for measurements of PDFF, this study revealed significant correlations between the PDFF of paraspinal muscles and the PDFF of vertebral bodies in postmenopausal women. The present investigation suggests a potentially close and clinically relevant relationship between spatially and functionally related compartments at the level of the lumbar spine in terms of fat compositions. However, follow-up studies are needed to further evaluate such correlations in larger cohorts, particularly consisting of premenopausal women as well as male subjects, in order to further explore clinical value and reliability of the reported correlations in terms of a potential role as biomarkers. Moreover, the distinct biological causes for the observed correlations between the PDFF of paraspinal and vertebral bone marrow compartments go beyond the scope of the present study and have yet to be elucidated.

## Author contributions

TB, DK, and JK designed the study. MD, SS, AR, JS, MND, DW, SR, EK, and DF coordinated subject enrolment, image acquisition, and data storage. NS, MD, SS, JS, and TB were responsible for data analysis, NS and TB performed statistics. NS, SS, JK, DK, and TB were involved in literature research. NS, MD, SS, AR, JS, MND, DW, SR, EK, DF, ER, CZ, JK, DK, and TB drafted the manuscript. The study was supervised by ER, CZ, JK, DK, and TB. All authors reviewed the manuscript before submission.

### Conflict of interest statement

JK received speaker honoraria from Philips Healthcare. DK received grant support from Philips Healthcare. The remaining authors declare that the research was conducted in the absence of any commercial or financial relationships that could be construed as a potential conflict of interest.

## Abbreviations

BMF: bone marrow fat
BMI: body mass index
CT: computed tomography
CV: coefficient of variation
DXA: dual energy X-ray absorptiometry
ESL: left erector spinae muscle
ESR: right erector spinae muscle
FOV: field of view
LBP: low back pain
MFI: muscle fat infiltration
MRI: magnetic resonance imaging
MRS: magnetic resonance spectroscopy
NMD: neuromuscular disease
PDFF: proton density fat fraction
ROI: region of interest
SD: standard deviation
T2DM: type 2 diabetes mellitus
TE: echo time
TR: repetition time.
